# Incidence, severity and perceived susceptibility of COVID-19 in the UK CrossFit population

**DOI:** 10.1186/s13102-021-00318-9

**Published:** 2021-09-06

**Authors:** Athalie Redwood-Brown, Grant William Ralston, Jennifer Wilson

**Affiliations:** 1 School of Science and Technology: Sport and Exercise Sciences, Nottingham Trent University, Erasmus Darwin 244, Clifton Campus, Clifton Lane, Nottingham, NG11 8NS UK; 2 FiiT for Life Education Ltd, Derby, UK; 3 College of Science and Engineering: Sport and Exercise Science, University of Derby, Kedleston Road, Derby, DE22 1GB UK

**Keywords:** COVID-19, CrossFit™, Functional exercise, Health, Physical activity

## Abstract

**Background:**

Contemporary literature indicates that a higher body mass index (BMI) serves as a risk factor for metabolic disease and is also correlated with greater disease severity. Subsequently, it has been linked to increased COVID-19 severity. The purpose of the study was to investigate whether regular CrossFit™ participation was associated with lower BMI, decreased COVID-19 severity and susceptibility.

**Methods:**

A cross-sectional study was conducted on 1806 CrossFit™ (CF) participants. Participants were asked about their age (yrs), sex (male vs. female), ethnic group, body height (cm) and weight (kg). Body mass index (BMI, kg/m^2^) was computed and consistent with WHO (2018) criteria. Participants self-reported their training history, health and lifestyle history, nutritional customs, present training status and suspected levels of exposure to COVID-19. Once submitted the collected data were coded, cleaned and analysed.

**Results:**

The final model comprised of 1806 CF individuals from an online survey response rate of 2086. The participants age ranged from 18 to 65+ yrs. Self-reported mean body mass index (BMI: kg/m^2^) reported that < 1% were underweight, 41% were healthy, 46% overweight, 10% class I obese, 2% class II obese, and < 1% class III obese. A Kruskal–Wallis H test compared gender and self-reported probability of being infected with COVID-19 with significant differences between subgroups (x^2^ (4, N = 1739) = 10.86, *p* = 0.03). Analysis of BMI and perceived severity of COVID-19 revealed a difference however not, significant (x^2^ (4, N = 1739) = 9.46, *p* = 0.051). Results on BMI and perceived probability of COVID-19 infection revealed no significant difference (x^2^ (4, N = 1739) = 2.68, *p* = 0.61). A separate analysis on BMI and perceived COVID-19 susceptibility revealed no significant difference (x^2^ (4, N = 1740) = 6.02, *p* = 0.20).

**Conclusions:**

The purpose of the study was to establish whether habitual CrossFit™ participation is associated with reduced BMI, and to further investigate whether habitual participation impacted perceptions of disease. Results of the study indicate that self-reported CrossFit™ participation during the first UK lockdown, measured in minutes of exercise was indicative of a lower BMI. This has been associated with greater host immunity to disease. A history of CrossFit™ participation was not shown to impact perceptions of disease. However, our sample population reported few changes to habitual exercise during lockdown which may be due to the ‘community’ and increased adherence associated with CrossFit™.

## Background

CrossFit™ was established in the year 2000 and is considered a form of high-intensity functional training (HIFT) with a global reach of 10,000 affiliates [6]. It is distinguishable from other forms of HIFT due to a broad focus across multiple fitness domains (see Table 1). As such, it promotes physical competency across the breadth of the fitness continuum, rather than facilitating specialisation.

**Table 1:** The 10 domains of fitness

Cardiovascular endurance	Stamina
Strength	Flexibility
Power	Speed
Coordination	Agility
Balance	Accuracy

Glassman [13] and Claudino et al. [6]

An essential aspect of CrossFit™ is its focus on preventative medicine and tackling chronic disease [25]. One of the primary theories that underpins the fitness methodology stems from the illness-wellness-fitness continuum [13]. This continuum spans sickness-wellness-fitness, where nearly every measurable value of health can be placed upon it. For example, a blood pressure (BP) measure of 160/95 mmHg is considered pathological (illness), 120/80 mmHg is considered normal or healthy (wellness) and lower ranges of BP are considered consistent with athletic populations (fitness) [34]. We can observe similar patterns for cholesterol, heartrate, bodyfat (BF) percentage, body mass index (BMI), etc. [13]. To this end, competence across all of fitness domains is considered to offer some health protection. Thus, individuals on the fitness end of the continuum need to pass through wellness before they get sick.

Key metrics have been used to measure an individual’s health protection [21]. One such example is body mass index, or BMI which is frequently used due to the ease of measurement. Literature reports a strong relationship between regular physical activity and a healthy BMI, and therefore those who are more physically active tend to demonstrate a lower BMI [10]. In the physical activity domain, authors note that ‘combined’ methods of resistance and aerobic (CRAE) training, such as CrossFit™, have a greater effect on reducing obesity than other forms of physical activity [1]. This has been associated with reduced risk of disease. Subsequently, those with a lower BMI are more likely to be at the wellness-fitness end of the aforementioned continuum. Individuals with a high BMI (particularly a BMI > 35) may find themselves toward the sickness end according to the sickness-wellness-fitness continuum. Therefore, it is no surprise that high BMI is one of the key indicators of medical intervention when fighting disease such as Coronavirus or COVID-19.

The Coronavirus Disease 2019 (COVID-19) has led to a worldwide research effort to identify specific population groups at greatest risk of intensive medical intervention when contracting the disease. Emerging evidence on poor health outcomes suggests that individuals with comorbidities including diabetes, cardiovascular disease (including hypertension), respiratory or kidney disease, specifically in older individuals are at greater risk [40]. Pulmonary function studies have also reported that obesity may be associated with reduced lung volume [19, 37]. Consequently, impaired lung function and immune response [17] impacts on the severity of COVID-19.

Several studies have further specified that individuals who are classified as clinically obese (BMI > 35) are more susceptible to COVID-19 and that it has the greatest impact on disease severity [24, 27]. Obesity has been reported to increase cardiovascular risk factors, and patients with such medical concerns are more at risk for severe COVID-19 infection and greater residue severity than healthy individuals [23, 27]. Obesity or excess ectopic fat deposition is also strongly correlated with individuals developing diabetes (or a high risk of diabetes), leading to impairment of insulin resistance and reduced cell function [15]. These factors lead to [in part] a reduction in the bodies capability to regulate insulin and, therefore, mediate the progression of COVID-19 [23]. Simonnet et al. [27] reported that obesity increases type 2 inflammation, which may impair immune response and the body’s innate ability to fight COVID-19.

Studies examining the risk factors associated with invasive medical interventions required to treat COVID-19 reported that a BMI > 30 was frequently present [24, 27]. Simonnet et al. [27] observed that disease severity increased with elevated BMI, specifically by 47.6% for individuals with a BMI greater than 30 and 28.2% for those with a BMI of more than 35. Simonnet and colleagues also reported that patients (85.7%) with a BMI of > 35 required invasive mechanical assisted ventilation during hospitalisation and were up to seven times more likely to require medical treatment than those with who had a BMI less than 25. Interestingly, other comorbidity risk factors including diabetes, cardiovascular disease, renal failure, stroke and hypertension related to COVID-19 severity were not independent of obesity [14, 22, 23, 27]. Nevertheless, many of these comorbidities and their related risk factors are negativity correlated with regular physical activity engagement and result in increased mortality in COVID-19 patients [40]. Subsequently, available evidence suggests that individuals who participate in regular physical activity may be at a reduced risk from developing severe COVID-19 symptoms. Several studies have also demonstrated a positive relationship habitual exercise and COVID-19 survival [4, 8, 32].

In order to reduce the spread and impact of COVID-19 many countries have enforced lockdowns. Most of these lockdown restrictions have limited the amount of physical activity conducted by individuals [16]. Lippi, Henry and Sanchis-Gomar, (2020) reported that acute cessation of physical activity facilitated the rapid onset of insulin resistance, muscle atrophy and a decrease in metabolic adaptations including a reduction in aerobic capacity and elevated blood pressure [3]. Examination of individuals exercise and physical activity levels in Norway have suggested that Norwegians have adopted a more sedentary lifestyle behaviour while engaging less in physical activity or exercise than they did prior to the ‘lockdown’ restrictions [16]. Subsequently, a number of authors such as Jiménez-Pavón et al. [18] have highlighted that continued physical activity is important during enforced lockdowns as it offers some protection against disease severity and promotes mental wellbeing. However, it should be acknowledged that an imposed lockdown may carry differing restrictions according to country and/or region, therefore any recommendations should be made in light of regional or national needs. To this end, the present study will focus on the UK CrossFit population.

## Aims

Contemporary literature indicates that health related metrics, such as BMI, serve as a significant risk factor to diseases such as COVID-19. It is also apparent that a lower BMI and habitual exercise can reduce the severity of disease thereby improving host resilience toward disease. To date, no authors have investigated whether perceptions of disease risk are impacted by habitual exercise. Subsequently, the paper aims are as follows:

i) To establish whether habitual CrossFit™ participation is associated with a reduction on health-related markers such as BMI.
ii) To examine perceptions of COVID-19 severity in habitual CrossFit™ participants.

## Methods

### Study design and settings

To investigate the effects of the United Kingdom’s (UK) nationwide lockdown restrictions on CrossFit™ (CF) participation and health-related markers (i.e., BMI). Furthermore, to examine the CrossFit™ participants perceptions of COVID-19 on self-reported susceptibility, severity and infection rates. A cross-sectional study was conducted on CrossFit™ participants using an anonymous online survey with participants being provided information on the general purpose of the study and their rights to anonymity. This online survey centred on a self-reported structured questionnaire and has been suggested by Geldsetzer [12] as an effective method to quickly reach a specific group of participants while safeguarding their safety under pandemic conditions. The questionnaire was made available via several social media platforms (i.e., CrossFit™ Facebook and Instagram) and from the CrossFit™ affiliate database (n = 650 CrossFit™ affiliates) for a period of 30-days period between 11th May 2020-to-10th June 2020. The time needed to complete the survey took approximately 10 min. Collected data were coded and processed anonymously.

### Study participants

**Fig. 1:**
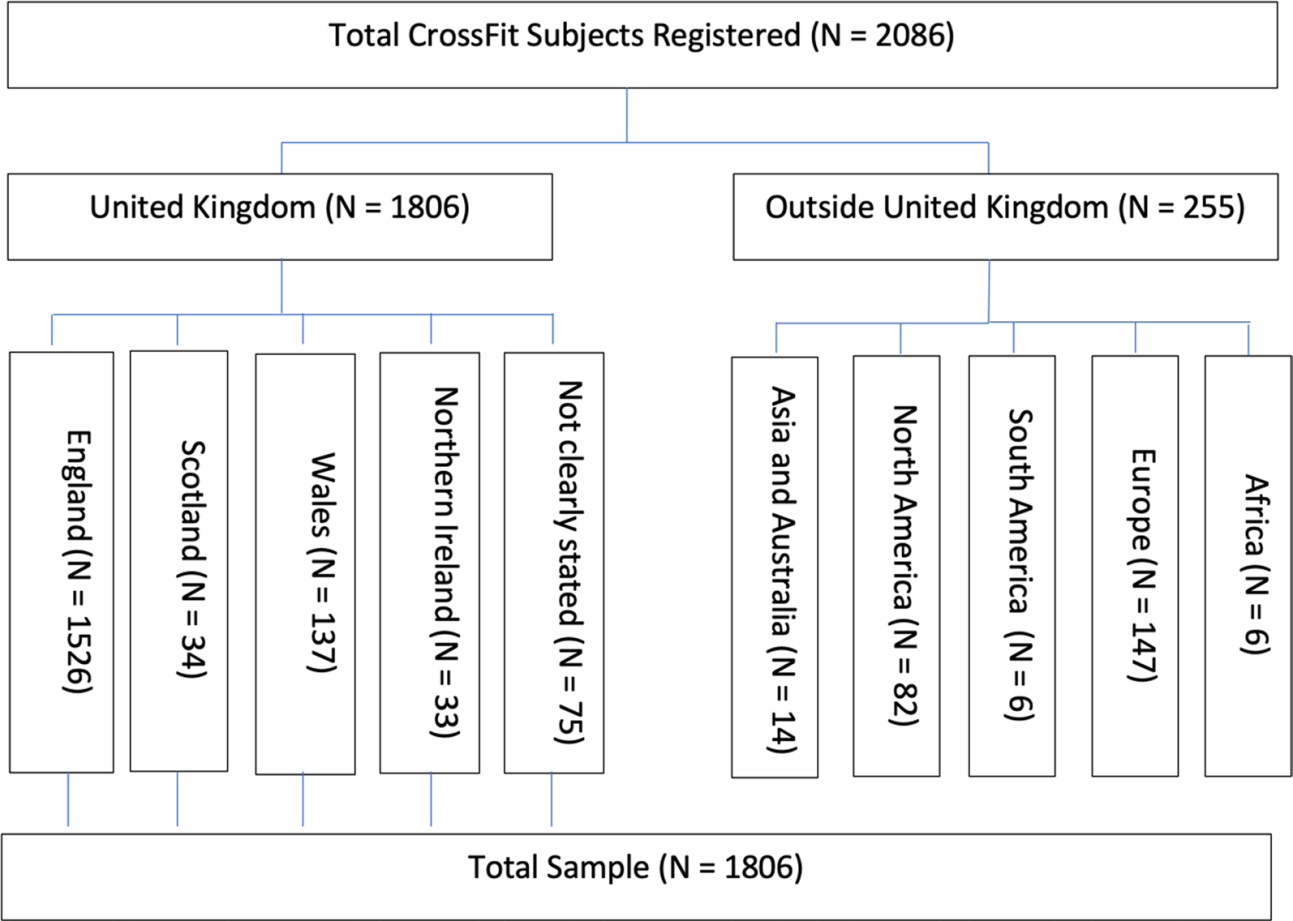
Flow chart of study sampling

A total of 2086 CrossFit™ participants registered to participate in the survey, including 1806 (86%) from within the UK. Regionally, participants in the survey resided from England (1526 [73%]), 34 (2%) from Scotland, 137 (7%) from Wales, and 33 (2%) from Northern Ireland. Seventy-five participants identified that they were from the UK but did not clearly specify with a further 255 (12%) stating they were from outside the UK (Fig. 1). Surveys were removed if they were either incomplete or not completed by someone residing in the UK. The representative sample population were male and female aged 18-to- ≥ 65 years (yrs) and have been participating in CrossFit™ for < 1-to-5 ≥ yrs.

### Assessment and measurements

We intentionally selected the four countries of the United Kingdom (England, Scotland, Wales and Northern Ireland) to give consistency to the findings. An online questionnaire was created via the platform survey monkey and participants registered their interest in the study via an online survey link. This platform was chosen due to the ease of dissemination to the population in question. The questionnaire was created using the survey tool and guidance information created by the World Health Organisation (WHO, 2020). Participants were asked to answer a series of questions relating to their medical history using a self-report method as suggested by Warburton et al. [36]. Basic demographics (age, sex) and health metrics (height and weight) were also reported to obtain BMI data. In line with the WHO guidance document this allowed for stratification of findings per population groups. In order to deem the training and fitness status of each respondent, training history and current training status was self-reported in line with previous research in this population group [20, 29].

Based on previous research and in line with the WHO guidance document, respondents were also asked questions pertaining to social demographics, risk perception (probability and severity) [2], perceptions of resilience [28] and lifestyle factors (e.g. stress and behaviour during lockdown) [28, 29]. As well as questions pertaining to their perceptions of risk of both contracting coronavirus and the perceived severity, their perceptions of the impact on their social circle was also included. Participants were also asked if they had or perceived they had, contracted coronavirus and the severity of the virus they experienced in line with the validated items created by Brewer et al. [2] on health behaviour and risk perception.

At the start of the questionnaire respondents were given details of the research process and purpose and asked for their informed consent prior to starting the questionnaire. The questionnaire was split into six sections including; general information, training, lifestyle, medical history, social circle, coronavirus experiences. Participants were informed that should they feel uncomfortable and want to abandon the survey—they can do so at any time prior to submission without consequence. Participants were also informed that all questions (beyond basic demographics) are optional—therefore if they feel uncomfortable answering a question, they can leave that question without consequence. At the end of the collection period all data was collected and downloaded into excel for cleaning and analysis purposes.

### Statistical analyses

#### Data analysis

Sample characteristics were described by frequency and percentage. Exploratory analyses were conducted to establish whether the data met parametric assumptions. To assess for data normality and homogeneity the Shapiro–Wilk and Levene tests were applied. According to Yap and Sim [39] the Shapiro–Wilk test is regarded as a powerful statistic for normality, with the Levene test applied to assess if variance was equal across groups. All the studied variables had a nonparametric distribution, except for exercise training experience. Following the Shapiro–Wilk and Levene test for homogeneity of variance, group analysis was completed using a non-parametric Kruskal–Wallis *H* test with rank sums tests post hoc. Descriptive statistics were also used to describe BMI ranking of participants by exercise training history, average minutes of exercise performed per week, CF training experience, number of CF classes performed per week. Statistical significance was tested with the Kruskal–Wallis *H* test for polytomous variables with Bonferroni correction for pairwise comparisons. The effect size (ES) was calculated using epsilon squared for the Kruskal–Wallis H test Epsilon squared was calculated as [33]:

$$\frac{H}{{(n^{2} - 1)/(n + 1)}}$$

ES of epsilon squared was classified as negligible (0.00–0.01), weak (0.01–0.04), moderate (0.04–0.16), relatively strong (0.16–0.36), strong (0.36–0.64) and very strong (0.64–1.00). Collected data were analysed using Statistical Package for Social Sciences (SPSS) version 27.0 (IBM Corp, Armonk, NY, USA). Significance levels for both main effects and post analyses was set at *p* = 0.05.

### Ethical consideration

The study protocol was reviewed and approved by Nottingham Trent Universities Non-Invasive Ethics Committee.

## Results

### Participants’ characteristics

A total of 2086 completed on-line surveys were received. The response rate was high with approximately 1% of the items required for scoring absent. Subscale values were computed only if more than 50% of the relevant items were valid with missing values either replaced by the subscale mean (Beglin and Fairburn 1992), otherwise cases were excluded. The final model comprised of 1806 individuals from a survey response rate of 2086 (Table 2). This total amount of 1806 was after exclusion of participants if they lived outside the United Kingdom. The participants age ranged from 18 to 65+ yrs with the sampled population including 7.0% of 18–24 yrs, 43% of 25–34 yrs, 32% of 35–44 yrs, 15% of 45–54 yrs, 3.1% of 55–64 yrs, and 0.4% of 65+ yrs. Ethnicity of the sample comprised of 95% white, < 1% black, 2% mixed race, and 2% other. Self-reported mean body mass index (BMI: kg/m^2^) reported that < 1% were underweight, 41% were healthy, 46% overweight, 10% class I obese, 2% class II obese, and < 1% class III obese. Self-reported fitness characteristics including exercise training history, average minutes of exercise performed, CrossFit™ training experience and number of CrossFit™ classes participated in per week prior to lockdown have been summarised in Table 3.

**Table 2:** Sample characteristics of CrossFit™ participants

Total (*n* = 1806)	N	%
Gender		
Men	799	44
Women	1006	56
Age (yrs) mean ± SD (min–max)		
18–24	127	7.0
25–34	778	43
35–44	571	32
45–54	266	15
55–64	56	3.1
65+	8	< 1
Ethnicity		
White	1721	95
Black	10	< 1
Mixed, n (%)	40	2
Other, n (%)	35	2
Height (1.69 m [SD ± 0.03])		
Weight (86.4 kg [SD ± 23.9])		
Body mass index (29.2 kg/m^2^ [SD ± 7.9])		
Underweight, n (%)	2	< 1
Healthy weight, n (%)	749	41
Overweight, n (%)	826	46
Obese I, n (%)	185	10
Obese II, n (%)	35	2
Obese III, n (%)	9	< 1

N, sample size; %, percentage; yrs, years; SD, standard deviation; m, metres; kg, kilograms

**Table 3:** Fitness characteristics of CF participants to BMI ranking

Characteristics	Underweight N (%)	Healthy weight, N (%)	Overweight, N (%)	Obese I, N (%)	Obese II, N (%)	Obese III, N (%)	Total N
Exercise training history (years) (%)							
< 1 yr	0 (0)	63 (45.0)	50 (35.5)	14 (10.0)	12 (8.5)	2 (1)	141
1–2 yrs	0 (0)	99 (43.0)	95 (41.1)	30 (13.0)	6 (2.6)	1 (< 1)	231
2–3 yrs	2 (< 1)	133 (42.1)	143 (45.3)	31 (9.8)	5 (1.6)	2 (< 1)	316
3–4 yrs	0 (0)	78 (40.8)	88 (46.1)	23 (12.0)	2 (1.0)	0 (0)	191
4–5 yrs	0 (0)	54 (41.2)	66 (50.4)	12 (9.2)	1 (< 1)	1 (< 1)	131
5+ yrs	0 (0)	310 (40.6)	373 (48.8)	70 (9.2)	8 (1.0)	3 (< 1)	764
Average minutes of exercise performed per week (%)							
< 60 min	0 (0)	2 (10)	5 (25)	3 (15)	10 (50)	0 (0)	20
60–119 min	1 (1.4)	27 (37.0)	32 (43.8)	8 (11.0)	3 (4.1)	2 (2.7)	73
120–179 min	1 (< 1)	187 (34.9)	226 (42.2)	120 (22.4)	0 (0)	2 (< 1)	536
180–240 min	0 (0)	137 (24.4)	357 (63.5)	66 (11.7)	0 (0)	2 (< 1)	562
> 240 min	0 (0)	384 (43.8)	422 (48.1)	58 (6.6)	10 (1.1)	3 (< 1)	877
CrossFit training experience (years) (%)							
< 1 yr	0 (0)	152 (45.2)	134 (39.9)	34 (10.1)	14 (4.2)	2 (< 1)	336
1–2 yrs	1 (< 1)	203 (44.5)	199 (43.6)	43 (9.4)	8 (1.8)	2 (< 1)	456
2–3 yrs	1 (< 1)	160 (41.7)	174 (45.3)	40 (10.4)	5 (1.3)	4 (1.0)	384
3–4 yrs	0 (0)	79 (38.5)	103 (50.2)	20 (9.8)	2 (1.0)	1 (< 1)	205
4–5 yrs	0 (0)	45 (38.5)	58 (49.6)	12 (10.3)	2 (1.7)	0 (0)	117
5+ yrs	0 (0)	95 (34.7)	146 (53.3)	31 (11.3)	3 (1.1)	0 (0)	274
Number of CrossFit classes performed per week							
1 per week	0 (0)	11 (28.4)	19 (50.0)	6 (15.8)	1 (2.6)	1 (2.6)	38
2 per week	1 (< 1)	57 (42.5)	56 (41.8)	16 (11.9)	4 (3.0)	0 (0)	134
3 per week	1 (< 1)	220 (44.2)	197 (39.6)	60 (12.0)	17 (3.1)	3 (< 1)	498
4 per week	0 (0)	195 (28.1)	255 (48.8)	51 (10.0)	8 (1.6)	3 (< 1)	512
5 or more per week	0 (0)	253 (42.8)	286 (48.4)	46 (7.8)	4 (< 1)	2 (< 1)	591

Prior to performing any further analysis, the Shapiro–Wilk and Levene's tests were applied to examine the necessary assumptions. The Shapiro–Wilk test for the distribution of the study variables specified that the study variables were not normally distributed (Table 4). The Levene's test was used to predict the homogeneity of the error variances (Table 4). The results of the Levene's test showed that the homogeneity assumption of variances was rejected. Therefore, as the assumptions were violated, requiring the performance of Kruskal–Wallis *H* test.

**Table 4:** Shapiro–Wilk normality test and Levene test

Group variables	Shapiro–Wilk normality test and Levene Test						
	Shapiro–Wilk	d.f	*p*-value	Levene	d.f.1	d.f.2	*p*-value
Exercise training experience	0.825	1796	< 0.001	2.483	5	1790	0.03
Minutes performed per wk	0.830	1796	< 0.001	0.848	5	1790	0.52
CF training history	0.877	1784	< 0.001	1.088	5	1778	0.37
CF classes per wk	0.862	1795	< 0.001	0.914	5	1762	0.47
Susceptibility of COVID-19	0.875	1740	< 0.001	0.136	4	1734	0.97
Severity of COVID-19	0.787	1739	< 0.001	1.055	4	1733	0.38
COVID-19 Infection	0.641	1669	< 0.001	1.408	4	1663	0.23

d.f., degrees of freedom; CF, CrossFit; wk, week; yrs, years

### Body mass index by self-reported sample characteristics

BMI ranking by sample characteristics is presented in Table 5. Results from the Kruskal–Wallis H test that compared the outcome of participants age range and BMI ranking reported a significant difference within subgroups (x^2^ (5, N = 1806) = 15.01, *p* = 0.01). Follow-up pairwise comparisons indicated that the age range of the participants and BMI ranking was significant between the following subgroups: 18–24 vs 25–34 yrs (*p* = 0.009), 18–24 vs 35–44 yrs (*p* < 0.001) and 18–24 vs 45–54 yrs (*p* = 0.002) respectively. Furthermore, when minutes of exercise performed per week were compared to BMI ranking (Table 5) a significant difference was found within subgroups (x^2^ (4, N = 1806) = 15.99, *p* = 0.003). Follow-up pairwise comparisons indicated that performing between 240 min of exercise per week compared to 180–240 min resulted in significant differences in participants BMI (*p* < 0.001). No other significant differences were found with BMI ranking and sample characteristics (ethnicity, *p* = 0.91; exercise experience, *p* = 0.96; CF training experience, *p* = 0.38; and number of CF classes per week, *p* = 0.12).

**Table 5:** BMI Ranking by Self-reported Characteristics of CrossFit™ Participants

	Descriptive statistics							
	Sample characteristics		BMI ranking					
	Total N	Mean [SD]	Total N	Mean [SD]	d.f	*H*	*p*	ES
Age	1806	2.53 [0.90]	1806	2.74 [0.76]	5	15.01	0.01^a^	0.01
Participant’s ethnicity	1806	1.11 [0.51]	1806	2.74 [0.76]	3	0.56	0.91	0.00
Exercise experience	1796	4.26 [1.77]	1806	2.74 [0.76]	5	0.99	0.96	0.00
Minutes of exercise per wk	1796	3.38 [3.38]	1806	2.74 [0.76]	4	15.99	0.003^a^	0.01
CF training experience [yrs]	1784	2.93 [1.62]	1806	2.74 [0.76]	5	5.31	0.38	0.00
CF classes per wk	1795	3.84 [1.04]	1806	2.74 [0.76]	4	7.38	0.12	0.00

BMI, body mass index; ES, effect size based on epsilon squared for Kruskal Wallis H test; n, number; SD, standard deviation; d.f., degrees of freedom; H, Kruskal–Wallis test; *p*, Kruskal–Wallis test probability value, CF, CrossFit; wk, week; yrs, years

^**a**^Superscript indicates significant differences between groups. Statistical significance: *p* < 0.05

### Self-reported probability, perceived susceptibility and severity of COVID-19

When asked about COVID-19 infection, a total of 1105 respondents (61.18%) reported that they had not contracted the disease. A small number of respondents (n = 15, 0.83%) reported testing positive for COVID-19, with a further 172 respondents (9.52%) reporting they suspected they had been infected with the disease. An additional total of 44 respondents (2.44%) reported testing negative for COVID-19 and 344 respondents (19.05%) reported that they could not confirm whether they had or hadn’t contracted COVID-19 by answering ‘don’t know’.

When asked about their immediate social circle, the majority of respondents did not know anyone in their immediate social circle who had contracted the disease (n = 1013, 56.09%). A total of 257 respondents (14.23%) reported knowing someone within their immediate social circle who had tested positive for COVID-19, whilst only 99 respondents (5.48%) reported knowing someone who received a negative test result. In addition, 289 respondents (16%) reported knowing someone who suspected they had COVID-19 without confirmation from testing. A total of 57 CrossFit™ affiliate owners completed the survey. Of this group, 53 reported that they had no confirmed deaths due to COVID-19 whilst four affiliate owners stated, ‘don’t know’.

Descriptive statistics presented in Table 6 outline participants’ perceptions of the COVID-19 disease. Self-reported perceptions of probability, susceptibility and severity indicate that a large proportion of the sample population did not perceive themselves to be at risk of infection or increased disease severity.

**Table 6:** Self- Reported COVID-19 Susceptibility, Severity and Infection to BMI

Characteristics	Underweight, N	Healthy weight, N	Overweight, N	Obese I, N	Obese II, N	Obese III, N	Total N
Self-reported probability of being infected with COVID-19							
Extremely unlikely	0	0	66	12	4	1	83
Unlikely	2	195	247	44	8	5	501
Somewhat unlikely	0	227	237	66	12	1	543
Likely	0	161	167	45	9	2	384
Extremely likely	0	33	27	8	1	0	69
Perceived susceptibility to COVID-19 (%)							
Not susceptible	0	135	155	25	6	2	323
A little susceptible	2	287	336	78	15	5	723
Somewhat susceptible	0	180	147	44	9	1	381
Susceptible	0	59	85	21	3	1	169
Very susceptible	0	21	20	7	1	0	49
Perceived severity to COVID-19 (%)							
Not severe	0	325	332	82	25	3	767
A little severe	0	217	255	59	2	5	538
Somewhat severe	1	116	110	20	5	1	253
Severe	1	17	34	12	0	0	64
Very severe	0	6	14	2	2	0	24
Infected with COVID-19							
Don’t know	2	141	140	47	9	3	342
No	0	434	522	105	20	4	1085
No, tested was negative	0	23	14	4	1	0	42
Yes, confirmed negative	0	80	68	16	4	2	170
Yes, tested positive	0	8	4	3	0	0	15

Probability, perceived susceptibility and severity of COVID-19 by sample characteristics (age, sex, ethnicity and BMI) is presented in Table 7. Results from the Kruskal–Wallis test that compared gender and the probability of being infected with COVID-19 indicated a significant difference subgroup (x^2^ (4, N = 1739) = 10.86, *p* = 0.03). Follow-up pairwise comparisons indicated that the self-reported probability of being infected with COVID-19 was significant between the following responses: ‘extremely likely vs somewhat unlikely’ (*p* = 0.03); ‘extremely likely vs unlikely’ (*p* = 0.03); likely vs somewhat unlikely (*p* = 0.02); likely vs unlikely (*p* = 0.021). No other significant differences were found when probability, perceived susceptibility and severity of COVID-19 was compared by sample characteristics (age, sex, ethnicity and BMI).

**Table 7:** Probability, perceived susceptibility and severity of COVID-19

Descriptive statistics							
Sample characteristics			Probability of being infected with COVID-19				
	Total N	Mean [SD]	Total N	Mean [SD]	Df	H	*p*-value
Age	1806	2.53 [0.90]	1739	2.81 [1.03]	4	7.74	0.10
Sex	1806	1.56 [0.50]	1739	2.81 [1.03]	4	10.86	0.03*
Ethnicity	1806	1.11 [0.51]	1739	2.81 [1.03]	4	2.50	0.64
BMI	1806	2.74 [0.76]	1739	2.81 [1.03]	4	2.68	0.61
CF Training history	1784	2.93 [1.62]	1739	2.81 [1.03]	4	5.24	0.26

Sample characteristics			Perceived susceptibility of being infected with COVID-19				
Age	1806	2.53 [0.90]	1740	2.33 [1.00]	4	2.75	0.60
Sex	1806	1.56 [0.50]	1740	2.33 [1.00]	4	6.82	0.15
Ethnicity	1806	1.11 [0.51]	1740	2.33 [1.00]	4	3.44	0.49
BMI	1806	2.74 [0.76]	1740	2.33 [1.00]	4	6.02	0.20
CF Training history	1784	2.93 [1.62]	1740	2.33 [1.00]	4	4.38	0.36

Sample characteristics			Perceived severity of COVID-19				
Age	1806	2.53 [0.90]	1739	1.80 [0.93]	4	3.44	0.49
Sex	1806	1.56 [0.50]	1739	1.80 [0.93]	4	3.91	0.42
Ethnicity	1806	1.11 [0.51]	1739	1.80 [0.93]	4	1.80	0.77
BMI	1806	2.74 [0.76]	1739	1.80 [0.93]	4	9.46	0.051
CF Training history	1784	2.93 [1.62]	1739	1.80 [0.93]	4	2.80	0.59

n, number; SD, standard deviation; H, Kruskal–Wallis test; *p*-value, Kruskal–Wallis test probability value

^*^Significant differences between groups

## Discussion

The purpose of the study was to establish whether habitual CrossFit™ participation is associated with reduced BMI, which is often considered a marker for increased risk of chronic disease and infection. The study further investigated participant perceptions of COVID-19 to understand whether fitness experiences impacted beliefs about susceptibility to disease. The survey considered key metrics relating to exercise history and training frequency, as well as perceptions of health and disease (with particular attention to COVID-19).

### Habitual CrossFit™ participation and BMI

Investigation of self-reported body composition indicated that BMI was not significantly associated with CrossFit™ training history. This was not unexpected given the stereotypical body type of individuals participating in CrossFit™ (especially at a high level). This type of training has been associated with mesomorph body types which can be misrepresented in a simple BMI calculation when using as a measure of “health” [7].

BMI is a recognised risk factor for disease that has gained significant interest due to the known relationship between obesity levels and disease severity [24, 27]. UK Government campaigns such as ‘Better Health’ have publicised an adverse relationship between BMI and diseases such as COVID-19, therefore despite the limitations of BMI as a measure, it exists to inform practitioners and individuals alike of the increased propensity toward disease.

In addition to training history, the number of CrossFit™ classes per week did not have a significant impact on BMI. Yet, there was a significant interaction between BMI and minutes of exercise per week. This was apparent between groups who exercised more than 240 min per week, and those who exercised between 180 and 240 min per week. The CrossFit™ model encourages a 4-day training cycle (3-day work, 1-day rest) [7] which in an average week, would see participants performing > 240 min of exercise per week. As expected, a large proportion of the sample population reported in excess of 240 min. This is particularly pertinent as authors consider frequent exercise to be integral to minimising viral infection and/or symptom reduction [21]. Thus, a large proportion of the sample population were demonstrating exercise habits which are associated with protection from chronic and infectious disease. We can only surmise that the key differences between number of classes and weekly training volume was due to the closure of facilities during the global pandemic, and therefore subsequent changes to training outside of the typical ‘class’ based structure.

Whilst these results do not differ from previous studies who have also investigated measures of body composition (for example, see: Suraki et al. [31]), our results provide a novel focus on participation during a period of national lockdown. Over 45% of participants reported that their exercise habits had not changed during this period, yet their sedentary behaviour during working hours had increased in a number of cases. Our report indicates that habitual exercise potentially offset the increased sedentary behaviour common to periods of lockdown [16]. Furthermore, over 50% of the population sampled reported no change to their mental state during the lockdown period, despite literature reporting that lockdown can have significant effects on an individual's mental health [18].

CrossFit™ favours a community-led approach and promotes social cohesion which is often regarded as a distinguishing feature of the training methodology [38]. CrossFit™ notably results in a greater sense of community and participants often report ‘higher levels of social capital and community belongingness’ which has a substantial effect on exercise adherence and mental wellbeing [38]. Compared to traditional forms of exercise, where participation is more likely to be an individual endeavour, CrossFit™ has demonstrated that community-based exercise is more likely to enhance adherence through developing social bonds between participants [38]. This sense of belonging could be argued as a key factor in maintaining mental well-being and habitual exercise during the lockdown period. CrossFit™ also places focus on the 10 fitness domains (Table 1) which require classes to be structured in a manner which incorporates a variety of training stimuli ranging from calisthenics to strongman style weightlifting [26]. Unlike other forms of HIFT, or combined resistance and aerobic training (CRAE), the CrossFit™ methodology encourages parity across the fitness domains to facilitate physical longevity and subsequently, move more individuals to the ‘fitness’ end of the illness-wellness-fitness continuum. Similar methods of CRAE and HIFT have been shown to have a greater effect on reducing metabolic risk factors associated with obesity, and result in improved aerobic capacity and activities of daily living (ADL) [1, 9, 35]. Yet, a key strength of the CrossFit™ methodology is its ability to retain and engage participants. Therefore, any exercise-related improvement to health behaviour is likely to be sustained.

### Perception, severity and susceptibility of COVID-19

A second aim of the paper was to investigate perceptions of COVID-19 severity and susceptibility within CrossFit™ participants. Evidence indicates that pandemic induced lockdowns typically result in reduced physical activity which can have harmful implications [11, 30]. Researchers have identified that staying physically active during induced lockdowns can have positive outcomes for mental and physical health. Therefore, exercise is considered as a mechanism for mitigating diseases such as COVID-19 [5]. To date, no authors have investigated how regular activity impacts perceptions of disease in habitually trained individuals. Therefore, we were particularly interested in investigating the perceptions of COVID-19 susceptibility and severity in habitually trained CrossFit™ participants. On a secondary level, the investigation also sought to identify whether participants considered their training history and self-reported body composition to impact their perceptions of disease.

Our results indicate that habitually trained CrossFit™ participants did not consider their training history to impact the probability of getting infected with COVID-19. This result was unexpected. Furthermore, perceived susceptibility toward the disease was not impacted by BMI classification, nor were perceptions of disease severity. Again, these results were unexpected. Given the campaigns, such as ‘Better Health’, which outline a relationship between BMI and disease severity, it was expected that participants would perceive a level of immunity to diseases such as COVID-19. This was not the case in the sample population, though the significance value demonstrates a trend toward perceptions of severity being ‘not severe’ (*p* = 0.051). Furthermore, descriptive statistics shown in Table 6 indicate that 42% of participants perceived disease severity to be ‘not severe’ and 30% of participants perceived disease severity to be ‘a little severe’. Whilst this trend in data is indicative of positive perceptions of disease severity, results were collected during the first UK lockdown. At this time, perceptions of the COVID-19 disease may have been based on a limited understanding of disease implications.

At the time of data collection, only fifteen individuals (0.8% of the sample) had contracted the COVID-19 disease and only 2 of these received minor treatment for the disease. Furthermore, a large proportion of the respondents (56.09%, n = 1013) reported that no one in their immediate social circle had contracted the disease. There is an implication that habitually CrossFit™ trained individuals did not perceive themselves or their immediate social circle to be at increased risk due to the low number of localised infections within CrossFit™ groups and social circles. As such, results collected from the first national lockdown were inconclusive. This implies that further investigation is necessary to understand whether a relationship between habitual exercise, health behaviour and perceptions of disease exists in the habitually trained.

### Study limitations

While this study provides a comprehensive examination of the impact of the COVID-19 pandemic on this population group, it does have several limitations. Firstly, this study examined self-reported information related to exercise levels before and during the initial lockdown. This feasibly may have led to over-reporting bias of participants physical activity levels and inaccurate self-reporting of body weight. Additionally, participants were recruited via social media and completed an online questionnaire, resulting in the sample being biased toward those who have access to and are familiar with technology. However, the overriding need for participant safety under pandemic conditions meant that the online survey was the most effective method of data collection. Furthermore, the usefulness and evidence of the recommendations are restricted due to the data being collected during the initial lockdown phase. At the time of data collection, limited data were available regarding the reporting of COVID-19 infection locally or nationally.

Longitudinal research is required to investigate the long-term effects of COVID-19 and this populations perception towards habitual exercise, health behaviour and COVID-19 severity. Due to not having a pre-pandemic (baseline) measure, it is difficult to make inferences about the impact COVID-19 has had on this population’s perception towards COVID-19 severity as lockdown restrictions continue. Longitudinal research that compares early lockdown data with data from subsequent time points could be valuable. This would help identify the continuing impact of COVID-19 as it develops, and the researchers are planning a follow-up study to address this. To this end, a follow-up study will be conducted to draw comparisons between the first UK lockdown, and most recent UK lockdown. From this, we will be able to identify whether exercise habits have been impacted by the prolonged UK lockdown, and whether this has changed perceptions of disease severity and susceptibility in habitually trained CrossFit™ participants.

## Conclusion

Historically, there are few authors who have investigated self-reported measures of health and wellness in habitually trained CrossFit™ participants. Hence, we were particularly interested to analyse the objective measures associated with disease. In light of the current pandemic, the investigation focussed on self-reported measures of body composition within habitually trained CrossFit™ participants. This did not account for other body composition factors such as fat free mass.

The documented relationship between BMI and COVID-19 infection found in previous studies has predominately investigated untrained individuals and therefore the ranges in BMI are substantial. It is widely accepted that regular exercise can reduce the risk of diseases associated with compromised immune function [21]. It is further acknowledged that *“a strong host immune response to COVID-19 is a key factor, for protection against infection and avoiding reaching severe stages of the disease”* (Ranasinghe et al. 2020). Thus, there is a strong argument that more individuals need to move to the fitness end of the sickness-wellness-fitness continuum, as proposed by Glassman [13]. It is recognised that those at the fitness end of the continuum are perhaps no less susceptible to disease, but a strong host immune response may allow for greater resiliency when fighting infection (Ranasinghe et al. 2020). Current investigations indicate that the immune response and protection from disease in the habitually trained is largely dependent on the format of exercise training (with particular focus on intensity, duration and type) [21]. The work of Laddu and colleagues further suggests that a larger review of exercise habits, health-related metrics and disease is necessary.

The purpose of the study was to establish whether habitual CrossFit™ participation is associated with reduced BMI, and to further investigate whether habitual participation impacted perceptions of disease. Results of the study indicate that self-reported CrossFit™ participation during the first UK lockdown, measured in minutes of exercise was indicative of a lower BMI. This has been associated with greater host immunity to disease. A history of CrossFit™ participation was not shown to impact perceptions of disease, particularly relating to probability of COVID-19 infection. However, a positive trend was observed between BMI ranking and perceptions of disease severity. Finally, our sample population reported few changes to habitual exercise during the induced lockdown, which may be due to the community and increased adherence associated with CrossFit™.

## Data Availability

The datasets used and/or analysed during the current study are available from the corresponding author (JW) on reasonable request. The data sets are not publicly available due to restrictions imposed by the Nottingham Trent University non-invasive ethics committee (to avoid compromising the privacy of research participants).

## Abbreviations

ADL: Activities of daily living
BF: Bodyfat
BMI: Body mass index
BP: Blood pressure
CF: CrossFit™
CRAE: Combined methods of resistance and aerobic training
HIFT: High-intensity functional training
